# The pattern of gene amplification of members of the Plasmodium vivax erythrocyte binding-like proteins family across the Amazon rainforest

**DOI:** 10.21203/rs.3.rs-7048749/v1

**Published:** 2025-07-10

**Authors:** Karla S. Guedes, Gabriela M. Fernandes, Bruno A. M. Sanchez, Francis B. Ntumngia, John H Adams, Flora S. Kano, Cor J. F. Fontes, Tais N. Sousa, Luzia H. Carvalho

**Affiliations:** Júlio Müller Hospital, Universidade Federal de Mato Grosso (UFMT); Instituto René Rachou, Fundação Oswaldo Cruz (Fiocruz); Universidade Federal de Mato Grosso (UFMT) – Campus Sinop; University of South Florida; University of South Florida; Instituto René Rachou, Fundação Oswaldo Cruz (Fiocruz); Júlio Müller Hospital, Universidade Federal de Mato Grosso (UFMT); Instituto René Rachou, Fundação Oswaldo Cruz (Fiocruz); Instituto René Rachou, Fundação Oswaldo Cruz (Fiocruz)

**Keywords:** Plasmodium vivax, malaria, copy number variation, invasion protein, DBP, EBP2, EBP/DBP2, DARC-positive genotypes

## Abstract

**Background:**

In *Plasmodium vivax* malaria, the Duffy Binding Protein (DBP), a key erythrocyte binding-like (EBL) protein, enables invasion of DARC (Duffy Antigen/Receptor for Chemokines) positive reticulocytes. Another EBL member, the erythrocyte binding protein 2 (EBP2, also known as EBP/DBP2), shares structural features with DBP, though its function in erythrocyte invasion remains unclear. While copy number variation (CNV) in EBL genes have been well-documented, data on *dbpand ebp2* amplifications in *P. vivax* isolates from South America remain scarce. This study investigates CNV in these two *ebl genes* across Amazon populations and examines their association with host DARC genotypes.

**Methods:**

A total of 192 *P. vivax* isolates from three malaria-endemic sites of the Amazon region (eastern, western, and southwestern) were analyzed. DARC polymorphisms were genotyped by real-time PCR with allele-specific oligonucleotides. CNV at *dbp* and *ebp2*loci was estimated by quantitative PCR (qPCR), using the *β-tubulin* gene as an internal reference. Gene copy numbers were stratified by geographical origin and host DARC genotype.

**Results:**

Amplification of *dbp* and *ebp2* genes varied across Amazon regions. In the western region, 25% of *P. vivax* isolates showed *dbp* gene amplification (up to 8 copies), compared to 2–9% in the southwestern and eastern regions (2–3 copies). Overall, *ebp2*amplification was less frequent, detected in 15% of *P. vivax* isolates from the western and in 1–4% from other regions. In the study areas, all individuals were DARC positives, and no association was observed between gene CNVs and DARC genotypes.

**Conclusions:**

This study identifies *dbp* and *ebp2*gene amplifications in *P. vivax* isolates from the Amazon rainforest, with regional CNV variation but no association with any DARC-positive genotype. These findings support further investigation into gene amplifications to elucidate their biological and immunological significance in DARC-positive populations.

## Background

The invasion of red blood cells by *Plasmodium spp*. is a complex biological process that requires the coordinated engagement of multiple parasite ligands with specific receptors on the erythrocyte membrane [1, 2]. In the case of *Plasmodium vivax*, the most widespread human malaria parasite [3], there are distinct ligand-receptor interactions that provide strict selectivity for reticulocytes [4–6]. The process of reticulocytes invasion by *P. vivax* depends on the Erythrocyte Binding-Like (EBL) proteins family [7] whose best-characterized member is the Duffy binding protein (DBP), which binds to the human atypical chemokine receptor 1 (ACKR1 also named Duffy antigen receptor for chemokines, DARC) [8]. The interaction between the ligand domain of DBP (region II, DBPII) and DARC receptor is central to blood-stage infection, as individuals lacking DARC on the surface of the RBCs (DARC-negative) exhibit significantly reduced susceptibility to *P. vivax* infection [9]. Thus, a vaccine that elicits antibodies able to block the DBPII/DARC interface would be expected to block parasite invasion (reviewed by [10]).

The long-standing hypothesis that *P. vivax* invades reticulocytes exclusively through the DBPII-DARC interaction has been increasingly challenged by numerous reports of *P. vivax* infections in DARC-negative individuals [11]. Towards understanding the mechanisms of *P. vivax* invasion in DARC-negative individuals, numerous studies have investigated other parasite ligands and alternative receptors (reviewed by [12]). In 2013, a notable study by Hester et al. [13], which involved de novo assembly of a field isolate genome, revealed novel *P. vivax* erythrocyte invasion genes. One of these genes coding a novel *P. vivax* DBP homolog, erythrocyte binding protein 2 (EBP2 also termed EBP/DBP2). EBP2 primary structure has the key conserved domain features characteristic of EBL protein family, including a DBL ligand domain (region II) considered essential for receptor recognition and merozoite invasion [7]. While some studies suggest that EBP2 binds preferentially to immature DARC-positive reticulocytes [14], others suggest that it binds to both reticulocytes and normocytes, independently of the DARC receptor [15]. Although the role of EBP2 in *P. vivax* reticulocyte invasion is still unknown, the combined antibody response to DBP and EBP2 has an additive effect in reducing the risk of *P. vivax* malaria in PNG children [16, 17].

The detection of gene copy number variation (CNV) in DBP and EBP2 added another layer of genetic complexity to *P. vivax* infections. In 2013, Menard et al. reported the first evidence of *dbp* gene amplification in *P. vivax* isolates from Madagascar, where infections occur in both DARC-positive and DARC-negative individuals [18]. The same authors detected *dbp* duplication in *P. vivax* infected blood samples from travelers returning from America, Africa, Asia and Melanesia. Subsequent studies confirmed that *P. vivax* strains carrying multiple copies of the *dbp* gene have a global distribution [19–24] and do not seem to have any association with the DARC negativity barrier [25, 26]. At this point, there is still no consensus about the role of *dbp* gene amplification in the *P. vivax* infection [12]. Several hypotheses have been proposed, including the possibility that gene amplification enables the parasite to evade the host immune response [26, 27].

In contrast to DBP, few studies have explored the genetic diversity of *ebp2* gene. Most available data is from Asian and, to a lesser extent, African populations [25, 28]. Recently, we have analyzed *P. vivax* isolates from the Brazilian Amazon region and found that the DBL domain of EBP2 is significantly less polymorphic than of DBP [29], a finding consistent with reports from other regions [13, 25, 28, 30]. Although studies on *ebp2* gene amplification remain scarce, available data suggest that gene multiplication is common in African populations, in both DARC-positive and DARC-negative individuals [22, 23, 25].

To gain insight into the gene amplification of the two main EBL proteins currently known to be involved in reticulocyte invasion (DBP and EBP2), this study assessed the copy number variation of the *dbp* and *ebp2* genes in *P. vivax* isolates from different endemic areas of the Amazon region, where malaria transmission ranges from hypoendemic to mesoendemic [31]. Considering that malaria transmission in the Amazon rainforest is associated with DARC-positive carriers, we further explore the correlation of gene copy number with DARC genotypes.

## Methods

### Ethical approval and consent to participate

The ethical and methodological procedures of this study were approved by the Human Research Ethics Committee of the René Rachou Institute / FIOCRUZ Minas (approval No. 4,041,859; CAAE 15632719.0.0000.5091), as well as by the Research Ethics Committee of the Júlio Muller University Hospital (approval No. 53041521.6.0000.5541). The study participants were provided with information regarding the objectives and procedures of the study, and their voluntary participation was requested and confirmed through written formal consent. In the case of child participants, written formal consent was obtained from their next of kin, caregivers, or guardians. Data were separated from personal identifiers through use of a code. All biological samples analyzed in this study were stored in the biorepository of the René Rachou Institute – FIOCRUZ Minas, under the following approved protocols: No. 07/2006, No. 07/2009, No. 12/2010, No. 26/2013, and No. 1.821.955/2016. The biorepository is curated by L. H. Carvalho and F. S. Kano.

The current study was conducted according to Laboratory biosafety and biosecurity policy guidelines of the Oswaldo Cruz Foundation (FIOCRUZ, Ministry of Health, Brazil (http://www.fiocruz.br/biosseguranca/Bis/manuais/biosseg_manuais.html).

### Study sites and sample collection

**Figure 1:**
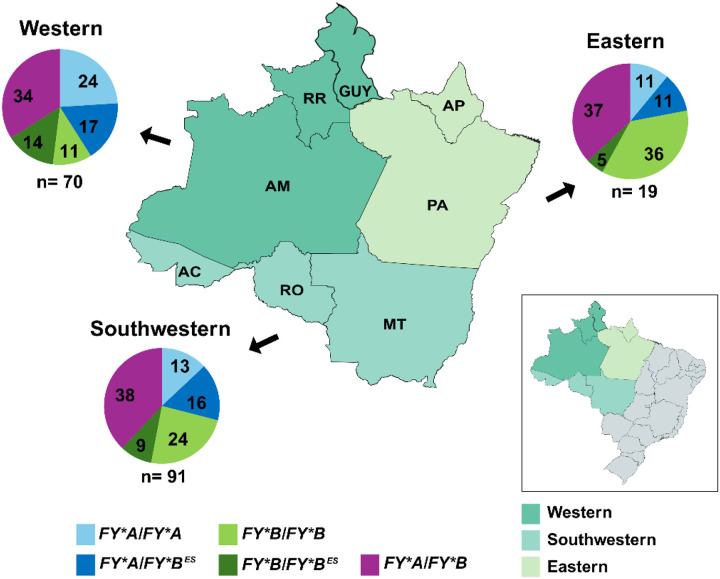
Distribution of DARC genotypes in the study population, stratified by malaria-endemic regions of the Amazon rainforest (western, southwestern, and eastern). Each pie chart indicates the genotype frequency per endemic site: Western (n = 70), Southwestern (n = 91), and Eastern (n = 19). Positive DARC genotypes were represented as FY*A/FY*A (blue) FY*A/FY*B^*ES*^ (dark blue), FY*B/FY*B (green), FY*B/FY*B^*es*^ (dark green), and FY*A/FY*B (purple). Regional divisions were mapped by state: western (AM, RR), southwestern (RO, MT, AC), and eastern (PA, AP); *P. vivax* samples from Guyana (GUY) were grouped under the western Amazon region. The inset illustrates the geographical position of these three regions in the context of other Brazilian states.

A total of 192 *Plasmodium vivax* field isolates were collected from patients across three regions of the Amazon, grouped as follows (Fig. 1, on the inset map): eastern Amazon — Amapá (AP, n = 4) and Para (PA, n = 19); western Amazon — Amazonas (AM, n = 32), Roraima (RR, n = 39), and Guyana (GUY, formerly British Guiana, n = 5); and southwestern Amazon — Rondônia (RO, n = 60), Mato Grosso (MT, n = 30), and Acre (AC, n = 3). The samples were collected between 2017 and 2023. Blood samples (5 mL) were obtained via venipuncture using EDTA as an anticoagulant, following written informed consent from all study participants.

### Malaria diagnosis

At the time of blood collection, all individuals were submitted to a finger-prick for malaria diagnosis by light microscopy. The Giemsa-stained thick blood smears were prepared and examined by experienced local microscopists, according to the malaria diagnosis guidelines of the Brazilian Ministry of Health. Genomic DNA was extracted from whole blood samples collected in EDTA, using the Puregene blood core kit B (Qiagen, Minneapolis, MN, USA), according to manufacturers’ instructions. Species-specific real-time PCR was performed as previously described [32].

### DARC genotyping

Two TaqMan assays (Applied Biosystems, USA) were used to genotype major DARC polymorphisms: the T-33C substitution in the RBC-specific GATA1 transcription factor binding motif (rs2814778, ID assay reference: C_15769614_10), and the G125A polymorphism in exon 2 (rs12075, ID assay reference: C_2493442_20). The amplification reactions were carried out on a ViiA7 Real-Time PCR System (Applied Biosystems, USA), using 2.5 μL of TaqMan^®^ 2x Universal PCR Master Mix, 0.25 μL of the specific TaqMan^®^ SNP Genotyping Assay, 1.25 μL of DNase and RNase-free water, and 1 μL of genomic DNA (~ 10 ng/μL). Each assay included both negative controls (water in place of DNA) and positive controls, consisting of previously genotyped samples, as previously described [33]. Data analysis was performed using QuantStudio Real-Time PCR Software v1.3.7 (Applied Biosystems, USA).

### Relative quantification of dbp and ebp2 copy number

Gene copy number was estimated by quantitative PCR (qPCR), following the optimized protocol of Roesch et al. (2018) [25]. The *β-tubulin* gene was used as an internal reference. qPCR reactions were performed in 10 μL total volumes, containing 5 μL of GoTaq^®^ qPCR Master Mix 2X (Promega), 2.2 μL of DNase and RNase-free water, and 1 μL of template DNA. Primer concentrations were as follows: 900 nM for *dbp* (CN_DBP_F and CN_DBP_R); 300 nM for *ebp2* (CN_EBP2_F and CN_EBP2_R) and *β-tubulin* (CN_3-tubulin_F, and CN_ β-tubulin _R) (Table S1).

Amplification and fluorescence detection were carried out on a ViiA7 Real-Time PCR System (Applied Biosystems, USA), using the following cycling conditions: 50°C for 2 minutes, 95°C for 10 minutes, followed by 40 cycles of 95°C for 15 seconds and 60°C for 1 minute, with a final step of 95°C for 15 seconds. All reactions were run in triplicate on 96- or 384-well plates. The copy numbers were estimated relative to a standard curve by using synthetic genes construct (gBlock) containing a single copy each of *β-tubulin, dbp*, and *ebp2*, on a 1:1:1 ratio, designed by Integrated DNA Technologies, Inc. (IDT, USA, 2023). Two commercial plasmids, pIDTSMART-AMP, also synthesized by IDT, were used for optimization: one containing *dbp* and *β-tubulin* inserts (1:1), and the other with *ebp2* and *β-tubulin* (1:1). Data were processed and 2^−ΔΔCT^ method (where CT is the cycle threshold) was used to determine the number of copies of each sample. A positive control consisting of an isolate previously validated to carry one copy of each gene (confirmed by independent methods) and a negative control (water in place of DNA) were included in all runs. Replicates with a standard deviation greater than 0.3 or with CT values above 32 were excluded from analysis. Samples showing copy number variation were re-tested in independent experiments for confirmation. Isolates with estimated copy numbers below 0.5 were discarded. Final copy number values were rounded as follows: 0.5–1.4 to 1 copy, ≥ 1.5–2.4 to 2 copies, ≥ 2.5–3.4 to 3 copies, ≥ 3.5–4.4 to 4 copies, ≥ 4.5–5.4 to 5 copies, ≥ 5.5–6.4 to 6 copies, ≥ 6.5–7.4 to 7 copies, and ≥ 7.5–8.4 to 8 copies. Reactions were analyzed using QuantStudio Real-Time PCR Software v1.3.7 (Applied Biosystems, USA).

## Statistical analysis

A database was created using EpiData software (www.epidata.dk). The graphics and the statistical analyses were conducted using GraphPad Prism version 9.5.0 (GraphPad Software, La Jolla, CA, USA) and Stata software version 12.0 (StataCorp, College Station, TX, USA. Data normality was evaluated using the Shapiro–Wilk test. For comparisons of non-parametric continuous variables, the Kruskal-Wallis test was employed. Categorical variables were analyzed using the Chi-square test or Fisher’s exact test, as appropriate. Two-sided p-values < 0.05 were considered statistically significant. All data necessary to replicate the study’s findings are openly available (Table S2).

## Results

### Molecular confirmation of P. vivax infection and DARC genotypes in the study population

Of the 192 *P. vivax* infections detected by microscopy in this study, 182 (92%) were confirmed as *P. vivax* monoinfections by a species-specific PCR assay, with 10 (5%) of them identified as mixed infections with *P. vivax* and *P. falciparum* (Table S2). Most *P. vivax* patients included in the study were male (n = 146, 76%), with a median age of 36 years (IQR 26–45 years). In the study area, all individuals were DARC positive, with a predominance of FY*A/FY*B genotype in all Amazon regions (34–38%) (Fig. 1). DARC positive homozygous (FY*A/FY*A and FY*B/FY*B) and DARC-null positive genotypes (FY*A/FY*B^*ES*^ and FY*B/FY*B^*ES*^) were also detected in high frequencies in the study area (Fig. 1). Except for the genotype FY*B/FY*B, there were no significant differences between the Amazon regions (Table S3).

### dbp and ebp2 gene copy number variation in the Amazon

**Figure 2:**
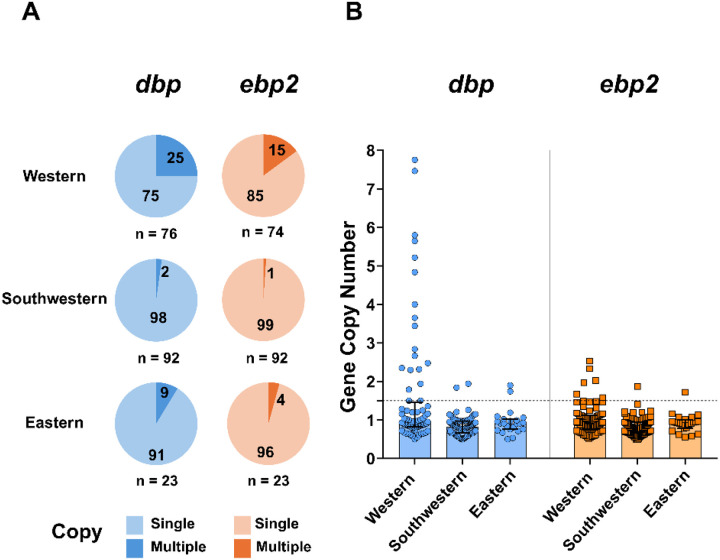
Distribution of *dbp* and *ebp2* gene copy number variations across Amazon Regions (eastern, western, and southwester). **(A**) Pie charts illustrate frequencies of single and multiple copies of *dbp* (light/dark blue) and *ebp2*(light/dark Orange) genes in the studied areas. (B) copy number variation of *dbp (blue)* and *ebp2(orange)*, considering values above the dashed threshold line as indicative of *P. vivax* samples with two or more gene copies.

The copy number variation (CNV) in the *dbp* and *ebp2* genes among *P. vivax* isolates showed differences across the distinct regions of the Amazon. The western region showed higher CNV frequencies, with 25% (19 of 76) and 15% (11 of 76) of isolates harboring CNVs in *dbp* and *ebp2*, respectively (Fig. 2A). In contrast, in the southwestern region, *gene* duplication frequencies were detected at much lower numbers, ranging from 2–1% for *dbp* (2 of 91) and *ebp2* (single isolate), respectively. Low CNV frequencies were also detected in the eastern region for both genes (9 to 4%; Fig. 2).

In terms of region, only *P. vivax* isolates from the western region exhibited up to eight copies of the *dbp* gene (Fig. 2B), while those from the eastern and southwestern regions exhibited a maximum of three copies. For the *ebp2* gene, the western region presented an estimated copy number of approximately three, whereas isolates from the other regions showed up to two copies. It is noteworthy that 10 out of 26 *P. vivax* isolates exhibited concurrent amplification of the *dbp* and *ebp2* genes, while most of them (16 out of 26) occurred in only one of the genes, i.e., 13 for *dbp* and 3 for *ebp2* (Table S4).

**Figure 3:**
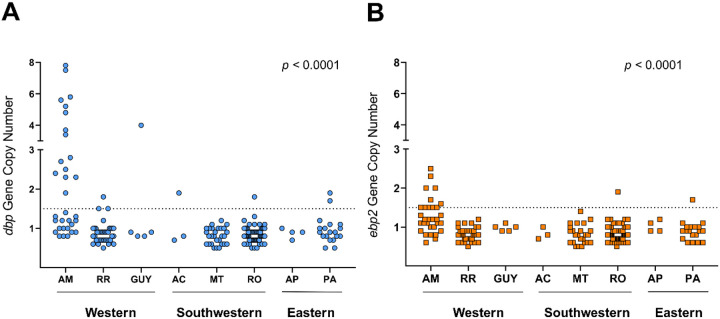
Distribution of *dbp* and *ebp2* gene copy number in *P. vivax* isolates from different states of Brazil and from Guyana. Copy number variation of *dbp* (A) and *ebp2 (B)*, considering values above the dashed threshold line as indicative of *P. vivax*samples with two or more gene copies. Amazon regional divisions were mapped as described in the legend of Fig. 1. Significant differences in *dbp* and *ebp2*gene copy number variation were observed between groups using the Kruskal–Wallis test (*p* <0.0001 for both genes). The state of Amazonas accounted for 65% (15 out of 23) and 85% (11 out of 13) of *P. vivax* isolates with *dbp*(A) and *ebp2*(B) gene amplifications, respectively.

At the local level, CNVs in *dbp* gene were detected in samples from Amazonas (AM), Roraima (RR), Para (PA), Guyana (GUY), Acre (AC), and Rondonia (RO), with AM presenting most of the gene multiplication (15 out of 23, 65%) (Fig. 3A). In contrast, *ebp2* CNV was restricted to isolates from AM, PA, and RO, with Amazonas accounting for 11 of 13 (85%) gene multiplications (Fig. 3B).

### Distribution of dbp and ebp2 gene copy numbers according to DARC genotype

**Figure 4:**
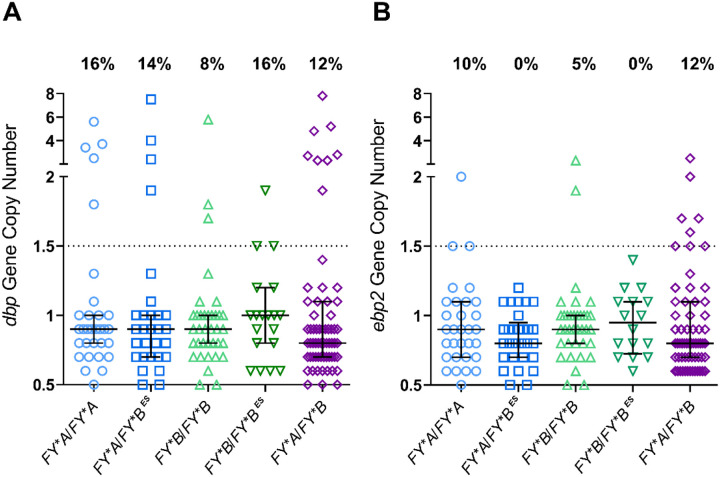
*dbp* and *ebp2* copy number in the study population distributed according to DARC genotype. Distribution of *dbp* (A) and *ebp2 (B)* gene copy number values in *P.vivax* patients with homozygous (FY*A/FY*A;FY*B/FY*B) or heterozygous (FY*A/FY*B FY*A/FY*B^*ES*^; FY*B/FY*B^*ES*^) DARC genotypes. The frequencies of multicopy genes for each DARC genotype are shown. For both *dbp* and *ebp2* genes, no significant differences were observed between groups (Table S5), nor between genotypes and the median copy number of each gene (Kruskal-Wallis test, p > 0.05 for all comparison).

Finally, it was investigated whether the occurrence and frequency of amplification of the *dbp* and *ebp2* genes could be associated with DARC genotypes. For this purpose, the gene amplification analysis stratified the total population according to host DARC genotype (Fig. 4). Taken together, the results demonstrated that neither *dbp* nor *ebp2* copy numbers were associated with any DARC genotype (Table S5, Fig. 4).

## Discussion

In different Amazon endemic settings, this study investigated copy number variation (CNV) in two *P. vivax* genes encoding proteins implicated in blood-stage invasion: *DBP*, a well characterized ligand that mediates invasion of DARC positive reticulocytes, and EBP2, a paralog with putative functional redundancy. The results of the gene amplification showed significant differences among the Amazon endemic sites, with the western region accounting for most parasites with multiple copies of the *dbp* or *ebp2* genes. Specifically, 25% of *P. vivax* isolates from the western region showed *dbp* gene amplification of up to 8 copies, compared to only 2–9% in the southwestern and eastern regions with 2–3 copies.. A similar pattern was observed for *ebp2*, with 15% in the western versus 1–4% in other regions. Unfortunately, the limited data available on *dbp* CNV- and the absence of data on *ebp2-* in the Brazilian Amazon region preclude any comparison of our findings with previous studies in the region. Despite of that, multiple copies of DBP were detected in 10% of *P. vivax* isolates from urban areas of Acre state, southwestern Amazon region [20]. Taken together, these results from the Amazon support the current view that copy number variation in genes associated with *P. vivax* reticulocyte invasion seems to be a common phenomenon occurring on a worldwide scale [18, 20, 21, 23–25, 34].

In the current study, the reason for the different spatial distribution of *dbp* and *ebp2* copy numbers among the three *P. vivax*-endemic Amazon settings is not clear. It is unlikely that variation in the host DARC gene accounts for the observed regional differences in gene duplication. Although the distribution of FY*B/FY*B genotype showed slight variation between the three regions, the overall DARC genotype frequencies were similar across the three Amazonian regions, with a predominance of the FY*A/FY*B genotype; this pattern reflects a population structure influenced by both Amerindian and European ancestry components [35]. Notably, most *P. vivax* isolates from the western Amazon originated from a long-term *P. vivax*-exposed population (Rio Pardo, AM, Table S2) formed mainly by Amazonian natives previously characterized by high contribution of Native Amerindian ancestry [35–37]. In contrast, *Plasmodium vivax* isolates from the southwestern and eastern Amazon are mainly derived from migrants originating in non-endemic regions of Brazil. In these regions, malaria is predominantly an occupational disease, strongly associated with activities such as deforestation and illegal mining [38]. Consequently, multiple, not-mutually exclusive, factors may have contributed to the difference in the CNV profiles between the western and southwestern/eastern regions, including differences in the intensity of malaria transmission, historical parasite population dynamics and host genetics [21]. Future studies with more presentative samples of the Amazon basin should address this topic.

Given that our previous findings and those of others have shown that susceptibility to *P. vivax* clinical malaria is influenced by DARC genotypes [35, 39, 40], we further investigated whether gene copy number variation was associated with DARC genotype. In this DARC positive population, no association was found between *dbp* or *ebp2* gene amplifications and DARC homozygous or heterozygous genotypes. Similar findings were reported in *P. vivax* isolates from Cambodia and Ethiopia, where DBP duplication was not linked to any specific DARC-positive genotype [20, 26]. Despite this, in populations where DARC-positive and DARC-negative individuals coexist, it has been suggested that the proportion of single-versus multicopy variants of DBP or EBP2 may differ between these groups [21, 22, 34]. It seems to be a consensus that larger studies are needed to further elucidate the role (if any) of DARC in DBP/EBP2 copy number variations.

While DBP duplication has been extensively studied in parasites from diverse geographical regions (reviewed by [12]), EBP2 amplification has received far less attention [22, 23, 25]. In our study, *ebp2* duplication was detected across all three surveyed regions in the Amazon (4–15%), albeit at a lower frequency than *dbp* amplification (2–25%). Duplication events in *P. vivax* isolates were predominantly gene-specific, with 62% (16 of 26) of samples displaying amplification in only one of the two erythrocyte-binding genes. Compared to isolates from the Amazon region, *P. vivax* isolates from Asia and Africa typically exhibit much higher frequencies of *EBP2* gene duplications (19 to 56%) [22, 25]. In Ethiopia, where DBP and EBP2 duplications are common, EBP2 amplification (but not DBP) is more prevalent in DARC-positive (46%) than in DARC-negative individuals (21%) [22]. This observation is further supported by recent evidence from Central Africa, where most of *P. vivax* isolates from DARC-negative individuals exhibited more than two *dbp* copies in the absence of *ebp2* amplification [23]. This is of particular interest as we and others have demonstrated that EBP2 can bind strongly to DARC-positive reticulocytes and moderately to DARC-negative reticulocytes [14, 19].

The current study has limitations that should be consider on data interpretation. First, all individuals presented an acute symptomatic *P. vivax* infection, with no quantitative assessment of parasitemia in thick-blood smear. Consequently, we were unable to evaluate whether levels of parasitemia would influence the gene copy variation, as it has been the subject of considerable speculation [20, 22, 23]; to proper address this topic and avoid discrepant results, it would be more appropriate to evaluate *P. vivax* biomass, as it is underrepresented in parasite counts obtained from peripheral blood smears [41]. Secondly, the relatively small sample size per site, combined with a cross-sectional design and substantial variability in the timing of data collection, precluded any attempt to conduct a temporal analysis of *dbp/ebp2* CNVs over time. Longitudinal studies should address this topic. Finally, our qPCR approach estimates *dbp* and *ebp2* gene copy numbers based on the dominant clone in each isolate. Notwithstanding, qPCR assay has been a more feasible protocol that has been used in different studies [25, 34]. Although outside the scope of the current manuscript, we agree with others that next-generation sequencing or a digital PCR approach should be able to overcome this issue of multiclonal infections [23, 25].

## Conclusion

This study confirms the low but appreciable presence of *dbp* and *ebp2* gene amplification in *P. vivax* isolates across the Amazon rainforest where DARC- negativity is rare. The results of these gene amplifications showed significant differences among the Amazon endemic settings, but with no association with any DARC positive genotype. Although the functional significance of *DBP* and *EBP2* amplifications requires further investigation, this pioneering study examining amplification at these loci in the Amazon provides important insights that may support the development of vaccines targeting *P. vivax* parasites harboring multiple copies of these key invasion ligands.

## Supplementary Material

Supplementary Files

This is a list of supplementary files associated with this preprint. Click to download.

- Additionalfile1.docx
- Additionalfile2.xlsx
